# Total Endoscopic Thyroidectomy with Intraoperative Laryngeal Nerve Monitoring

**DOI:** 10.1155/2016/7381792

**Published:** 2016-06-20

**Authors:** Bin Lv, Bin Zhang, Qing-Dong Zeng

**Affiliations:** Qilu Hospital of Shandong University, Department of General Surgery, Jinan 250012, Shandong, China

## Abstract

*Objective*. To evaluate the clinical efficacy of laryngeal nerve (LN) monitoring (LNM) during total endoscopic thyroidectomy via breast approach, with emphasis on the identification rates for RLN and EBSLN and the incidence of RLN paralysis.* Materials and Methods*. This retrospective study included 280 patients who underwent endoscopic thyroidectomy with or without LNM. RLN and EBSLN were identified using endoscopic magnification in the control group, while they were localized additionally by LNM in the LNM group. Demographic parameters and surgical outcomes were analyzed by statistical methods. Patients in the control group were also stratified by the side of thyroidectomy to determine difference in left and right RLN injury rates.* Results.* All procedures were successfully conducted without permanent LN damage. The identification rates for RLN and EBSLN were high in the LNM group compared to those of the control group, and the risk difference (RD) of temporary RLN injury between two groups was 6.3%. The risk of damage was slightly higher for the left RLN than for the right RLN in the control group, which was performed by a right-hand surgeon.* Conclusion.* The joint application of LNM and endoscopic magnified view endows total endoscopic thyroidectomy with ease, safety, and efficiency.

## 1. Introduction

Thyroid surgery has long been considered an effective treatment option for malignant thyroid diseases. The fast evolvement of medical technologies and instruments, such as minimally invasive surgical techniques and ultrasonic scalpels, has enabled a smaller incision length or incision placement at hidden sites away from the neck, resulting in nonevident scars. Minimally invasive thyroid surgery ranges from minimally invasive video-assisted thyroidectomy (MIVAT) to complete endoscopic thyroidectomy, and, compared with conventional procedures, it offers superior esthetics, decreased postoperative pain, and shortened hospitalization time. Endoscopic thyroidectomy via chest-breast approach or areola of breast approach, although not strictly minimally invasive, is welcomed by beauty-conscious patients worldwide. However, injuries to the external branch of the superior laryngeal nerve (EBSLN) and recurrent laryngeal nerve (RLN) are still well-defined hazards in endoscopic thyroid surgery. Postoperative symptoms of hoarseness, dyspnea, and dysphagia primarily indicate RLN damage, which can also remain asymptomatic in some cases. The rate of permanent RLN paralysis and the incidence of transient RLN palsy after thyroidectomy have been reported as 0.3%–3% and 5%–8%, respectively [1]. EBSLN injury may decrease the range of vocal cord vibration frequencies, consequently affecting the capability to generate a higher vocal register and decreasing voice projection. This nerve injury reportedly occurs in up to 58% patients during dissection or ligation of the superior thyroid pedicle [2, 3]. Visualization of the laryngeal nerve (LN) during surgery will aid in decreasing the incidence of nerve injury to some extent [4]. To date, numerous techniques to enhance intraoperative LN identification, which require special instruments and additional procedures during neck surgery, have been introduced and improved, including the use of commercially available electromyography endotracheal tube (ETT) and paired nerve monitoring (NM) system. Several studies have reported that LN monitoring (LNM) during conventional thyroidectomy enables the reliable localization of both EBSLN and RLN, with improved neurological outcomes [5, 6]. However, to the best of our knowledge, the effectiveness of LNM during total endoscopic thyroidectomy via the breast approach has not been assessed. Therefore, we conducted this study to evaluate the clinical efficacy of LNM during total endoscopic thyroidectomy via the breast approach, with emphasis on the identification rates for RLN and EBSLN and the incidence of RLN paralysis.

## 2. Materials and Methods

### 2.1. Patients

In order to avoid bias due to variable complexity in the surgical technique and guarantee standardization of all patients, we adhere to strict inclusion criteria as follows: age ≤ 45 years, presence of benign thyroid nodules measuring ≤ 4 cm or malignant lesions measuring ≤ 2 cm in their largest diameter, absence of cervical lymphadenopathy, no history of previous neck surgery, no comorbidity of Hashimoto's thyroiditis (HT), and absence of extrathyroidal metastases. The medical records of patients who underwent endoscopic thyroid surgery via the areola of breast approach at Qilu Hospital of Shandong University between January 2010 and January 2014 were retrospectively reviewed for patient demographics, volume of the treated thyroid, dominant nodule size, surgical scope (lobectomy versus total thyroidectomy), mean duration of the critical operating procedure, and neurological complications. In our study, all procedures were approved by Medical Ethics Committee of Qilu Hospital of Shandong University. Patients were diagnosed by ultrasonography (USG) and thyroid function test and provided written consent to undergo this surgical method after receiving an explanation of the risks and benefits. Patients with malignancies received thyroid stimulating hormone (TSH) suppressive therapy after surgery. Hormone replacement therapy was administered to patients with benign nodules.

### 2.2. Surgical Technique

All patients underwent endoscopic thyroid surgery under general anesthesia. We have been administering a half-dose neuromuscular blocking agent during the induction of anesthesia at our department since 2010, while we have been employing intraoperative nerve monitoring and stimulation using the NIM® 3.0 (Medtronic Inc., Minnesota, USA) in most patients since 2012. For this, intracutaneous grounding electrodes were inserted in the right upper arm. A specialized ETT was accurately placed such that the surface electrode was in contact with the true vocal cords, and the integrity of the NM system was confirmed by tapping test. Then, three incisions were placed along the margins of areola. One was an observation hole at the medial border of the right areola for videoendoscopy and removal of the specimen, while two operation holes were at the bilateral areola borders. The 30° 10-mm endoscope (Olympus Inc., JPN) was inserted and subcutaneously advanced for a distance of 20 cm with 2.4% ropivacaine and 0.2% epinephrine previously impregnating the subcutaneous tunneling. A puncture path was prepared in the anterior cervical midline, 3 cm above the sternal notch, to insert the pulse-generated monopolar probe. The surgical team included an intermediate grade general surgeon and two assistants specialized in endocrine surgery, and all procedures were meticulously performed with thorough hemostasis. En bloc lobectomy was achieved under adequate surgical field exposure using specialized retractors.

### 2.3. Nerve Identification

We routinely visualize but do not seal the superior and inferior thyroid arteries until LNs are exposed. In the control group, RLN and EBSLN were solely identified using endoscopic magnification, while, in the LNM group, they were identified using magnification through the endoscope and the NM system at the time of nerve dissection or thyroid vessels sealing. Neural stimulating responses were recorded according to the four-step routine (V1: initial vagal stimulation; R1: initial RLN stimulation; R2: after thyroid lobectomy or central compartment lymphadenectomy, as well as V2). The cosine wave and audio feedback outputted by the monitor alerted us to RLN proximity during anatomical dissection, while an audible signal and corresponding twitch of the cricothyroid muscle indicated the integral function of EBSLN. In the control group, the initial and final LNs identification was accomplished by endoscopic magnification only, while, in the LNM group, the initial LNs identification was prior to neural stimulation and the final after neural stimulation.

### 2.4. Assessment of Nerve Function

Evaluation of vocal cord motion was standardized by performing laryngoscopy within 3 days before and after the surgery [7]. All evaluations were performed by a consulting head and neck surgeon. Any decrease in vocal cord mobility was identified as paralysis. Affected patients underwent a 1-year follow-up with the written consents, with visits scheduled at 1-month intervals. Temporary RLN injury was defined as vocal cord dysfunction that resolved within 6 months, while permanent RLN injury was defined as persistent vocal cord dysfunction for >6 months.

### 2.5. Data Collection and Statistical Analysis

Patient age, nodule diameter, volume of treated thyroid, and duration of the critical operating procedure are expressed as means ± standard deviations (SDs). The volume of treated thyroid was calculated for all patients using the following formula: volume = *f* × (*A* × *B* × *C*) × 10^−3^ mL. Here, *f* is the correction coefficient for the elliptic model, which is assigned *π*/6 = 0.524, and *A*, *B*, and *C* represent the maximal dimensions assessed by USG in two mutually perpendicular planes [8]. The duration of the critical operating procedure was defined as the time period from exposure of the affected lobe to en bloc removal. Cross-tabulation analysis with the *χ* ^2^ test or Fisher's exact test was performed to validate the comparability of the two groups. Differences in surgical data were analyzed using Student's *t*-test. A *P* value of <0.05 was considered statistically significant.

## 3. Results

In total, we reviewed the records of 282 patients with no history of voice problems resulting from vocal cord palsy, nodules, or polyps. One patient developed hoarseness due to arytenoid dislocation and exhibited full recovery finally. Two patients were excluded because they developed a fluctuating neck mass due to thermal damage of trachea and underwent prompt repair for tracheal rupture. Eventually, we included 156 patients in the control group and 124 patients in the LNM group. In the control group, 127 patients (81.4%) underwent hemithyroidectomy, with or without central compartment lymphadenectomy and 29 (18.6%) underwent total thyroidectomy, with or without unilateral or bilateral central compartment lymphadenectomy, resulting in 185 nerves at risk (NARs). In the LNM group, 92 patients (74.2%) underwent a hemithyroidectomy and 32 (25.8%) underwent total thyroidectomy, both combined with or without central compartment lymphadenectomy, and this resulted in 156 NARs. There were no differences with regard to age, sex, and the pathological condition between the two groups (Table 1, *P* > 0.05 for all). Furthermore, all cases exhibited a similar degree of technical difficulty, as assessed from the volume of the treated thyroid (*P* = 0.48), nodule size (*P* = 0.23), and extent of surgery (*P* = 0.15). Although suspected benign nodules (largest diameter: >2 cm) were verified to be malignant by frozen section analysis in some patients, leading to a deviation from our inclusion criteria, there was no significant impact on the comparability of the two groups (Table 2). Clinical examinations revealed that no case of permanent RLN paralysis existed. Neural traction, heat conduction, and other ischemic nerve lesions resulted in a temporary RLN paresis rate of 7.57% (14 of 185 NARs) in the control group and 1.28% (two of 156 NARs) in the LNM group. This incidence was equally distributed throughout the research period, with no apparent subjective bias (nine cases in the first 140 patients and seven in the remaining). As shown in Table 1, LNM is an added protection to RLN (odds ratio (OR): 0.16; 95% confidence interval (CI): 0.04–0.71; observed power: 0.87) which yields a risk difference (RD) of 6.3% between two groups (*P* < 0.01), while the duration of the critical operating procedure showed no such difference (*P* = 0.13).

With regard to the efficacy of intraoperative neuromonitoring, the visual identification rates (identifying nerve by endoscopic magnification only) for both RLN and EBSLN were not significantly different between the two groups when only endoscopic magnification was used (*P* > 0.05 for all). Although statistical analysis indicated a slight difference in the final identification rate of RLN of two groups (*P* = 0.03), it seemed to be of no clinical significance (97.4% versus 100%, *P* = 0.12). However, evidently greater number of EBSLNs, which were not previously detected, were identified under LNM (Table 3, *P* < 0.01). At the same time, the number of left RLN palsies was observed to be greater than that of right RLN palsies. Accordingly, the control group was further divided into right and left lobectomy groups, and the incidence of left-sided palsy was higher (*P* = 0.04), even though the difference was not obviously significant (Table 4).

## 4. Discussion

Endoscopic thyroidectomy was introduced at our institute in 2010, and we have gradually and comprehensively mastered the procedure since then. An average of 93 endoscopic thyroid operations a year were performed by us now. Intraoperative NM was introduced in 2012 and became a routine practice and a basic item included in the consent procedure before surgery; since then, an increasing number of patients was convinced to experience this new nerve and muscle stimulation technique, which helped us in completing our retrospective study. We evaluated the clinical efficacy of LNM during total endoscopic thyroidectomy via the breast approach and found a significant improvement in the RLN and EBSLN detection rates with this modality.

Intraoperative RLN identification has long been credited with the benefit of decreasing the incidence of postoperative nerve paresis. Because endoscopic surgery offers a small space for surgical manipulation, stringent safety measures against nerve exposure are essential. In the LNM group, we routinely perform neural mapping (probing for RLN) until the entire course of RLN is exposed, and the incidence of temporary paresis decreased from 7.6% to 1.3% (RD, 6.3%) in our study. However, these results are limited by the inclusion of a relatively small sample size and nonrandomized design. In addition, though cases suffering RLN permanent injury were equally distributed throughout the research period (nine cases in the first 140 patients and seven in the remaining), we have become more experienced with the endoscopic technique over time and the improved outcomes in the LNM group could be in part due to this experience. However, duration of surgery was not different and that nerve injury did not decrease over time in the control group, which would argue against this. A prospective study with a larger cohort, similar to the study by Lorenz et al. [9], is necessary to rule out confounding factors (body mass index (BMI) and neck length) by stratified analysis or multiple logistic regression analysis and further clarify the advantages of LNM for both patients and less experienced surgeons. Terris et al. reported that the current incidence of postoperative vocal cord palsy is very low (1%-2%), necessitating a sample of approximately 1000 patients to determine a 1% decrease in the nerve injury rate [10].

Loss of neuromonitoring signal (visual and audio feedback vanished) often alludes RLN injury, either transient or permanent one. Signal failing to be restored (no R2 and V2 signal or R2 ≤ 1/2 R1) in 20 min is deemed to be nerve palsy, and the operation is terminated in total thyroidectomy. If needed, a second surgery resecting the other affected lobe is performed until the former paralytic nerve is restored. In those cases, we only enrolled the initial operation data into our study. Signal restoring (R2 ≥ 1/2 R1) in 20 min commonly occurs and is deemed to be normal condition.

LNM facilitates the identification of nerves at each step of dissection and ligation, resulting in safe and easy endoscopic surgery without inadvertent ligature, clamping, or heat or suction injury in most cases. In addition, LNM aids in documenting the status of RLN, which can be used as a reference in case of secondary intervention. In fact, we also recommend LNM for patients with latent nonrecurrent LN associated with a retroesophageal right subclavian artery, those with previous neck surgery or irradiation, those with palpable lymphadenopathy, and those with nodules showing RLN invasion or compression on USG and requiring open thyroidectomy. However, young surgeons should utilize this technique as an adjunct to understand more about the complex anatomy of RLN rather than relying on it completely, because the cost of this procedure is not yet covered by the Chinese health insurance system.

EBSLN is also at risk of inadvertent injury during thyroidectomy, although clinical trials have shown a low injury rate [3]. It is routinely probed and dissected until a portion of nerve is exposed at our institute, with corresponding contraction of the cricothyroid muscle during stimulation used as an indicator of an intact EBSLN. In our study, the rate of EBSLN localization increased from 42.9% to 75.6% with LNM, which might decrease the incidence of superior laryngeal nerve- (SLN-) related complications. This finding is consistent with the findings of Barczyński et al. [2] who reported the effectiveness of SLN monitoring in open thyroidectomy, where the postoperative video stroboscopy and videokymography were used to indicate an intact EBSLN. However, the costs of determining SLN injury (either intraoperative nerve monitoring and stimulation or the postoperative video stroboscopy and videokymography) in our healthcare environment are high; furthermore, the diagnostic value of patients' subjective complaints is low, which impedes further investigations [11].

In the present study, we statistically evaluated the duration of the critical operating procedure, which included the procedure between exposure of the affected thyroid and en bloc removal, and found no significant difference between the LNM and control groups. However, we followed a four-step LNM procedure (V1-R1-R2-V2) to complete the most time-consuming and delicate process during lobectomy, that is, RLN dissection. The surgeons involved in these procedures had accumulated sufficient surgical experience, and this resulted in a decreased surgical duration, decreased blood loss, and few postoperative complications and compensated, at least in part, for the time consumed by LNM.

We also evaluated patients stratified by the side of thyroidectomy to clarify the suspected tendency of increased left RLN paresis in the control group and found no obvious significant difference. If certain unknown confounders are excluded, this phenomenon can be explained as follows. First, the left RLN travels within the tracheoesophageal groove (TEG), whereas the right RLN courses anterior to TEG. Therefore, the left RLN is relatively better protected from ligation and retractor-induced trauma during open thyroidectomy [12]. However, the opposite is true during endoscopic surgery because of a small surgical field, increased lobe traction due to deeper and longer course of the RLN on the left, and deeper dissection using an ultrasonic scalpel. Second, right-hand surgeons stand between the patient's legs and insert the ultrasonic scalpel through a hole created on the left. The medial margin of the right lobe is pulled outwards, and the right RLN is just located under the surface tissue, whereas the lateral border of left lobe was drafted toward the midline, and some tract-like tissues may stretch or compress nerve to the trachea, leading to more stretch injuries and corresponding temporary paralyses. Third, the six patients with left RLN damage exhibit a higher BMI or a short neck, leading to further bias. In addition, prospective studies are necessary to verify these speculations.

In conclusion, the results of our study suggest that LNM is a sensitive and nerve-specific tool that decreases the incidence of complications associated with SLN and RLN damage during minimally invasive thyroid surgery restricted to the central neck compartment, particularly when surgery is performed by inexperienced surgeons. It endows endoscopic thyroidectomy with ease, safety, and efficiency.

## Figures and Tables

**Table 1:** Demographic parameters and surgical data.

	Control group	LNM group	*P*
Demographic parameters			
Age (years)	30.33 ± 6.65	28.90 ± 6.53	0.21
Male (%)	7 (4.5%)	4 (3.2%)	0.76^a^
Female (%)	149 (95.5%)	120 (96.8%)	
USG findings			
Volume of treated thyroid (mL)	14.05 ± 8.57 (2.91–28.06)	13.31 ± 9.02 (2.53–29.01)	0.48
Dominant nodule diameter (cm)	1.81 ± 0.84 (0.4–3.5)	1.69 ± 0.87 (0.3–3.8)	0.23
Techniques employed			
Hemithyroidectomy (%)	127 (81.4%)	92 (74.2%)	0.15
Total thyroidectomy (%)	29 (18.6%)	32 (25.8%)	
Duration of surgery (min)	39.87 ± 5.39	40.77 ± 4.42	0.13
Postoperative follow-up by fibrolaryngoscope^b^			
Transient RLN injury	14	2	<0.01
Normal vocal cords condition	171	154	

USG: ultrasonography; RLN: recurrent laryngeal nerve; LNM: laryngeal nerve monitoring.

^a^Fisher's exact test.

^b^The number of nerves at risk, not the number of patients, was counted.

**Table 2:** Pathological findings in patients with and without intraoperative LNM.

Treated pathology	Control group	LNM group	*P*
Nodular goiter (%)	47 (30.1%)	35 (28.2%)	0.74
Thyroid adenoma (%)	20 (12.8%)	13 (10.5%)	
DTC (%)	89 (57.1%)	76 (61.3%)	
T1	84	70	
T2^a^	5	6	
Nx	28	31	
N0	24	17	
N1a	37	28	

DTC: differentiated thyroid carcinoma; T1-T2, Nx–N1a: pathological staging according to the 7th edition of the AJCC TNM staging system; Nx: no palpable cervical lymph node or no lymphadenectomy.

^a^Suspected benign nodules that were pathologically proven malignant, not fulfilling our eligibility criteria.

**Table 3:** Efficacy of LNM (identification of 341 NARs).

	Control group	LNM group	*P*
Initial visual ID of RLN (%)	179/185 (96.8%)	152/156 (97.4%)	0.76^a^
Final ID of RLN (%)	179/185 (96.8%)	156/156 (100%)	0.03^a^
*P*	—	0.12^a^	—
Initial visual ID of EBSLN (%)	73/185 (39.5%)	67/156 (42.9%)	0.51
Final ID of EBSLN (%)	73/185 (39.5%)	118/156 (75.6%)	<0.01
*P*	—	<0.01	—

ID: identification; NAR: nerve at risk; LNM: laryngeal nerve monitoring; RLN: recurrent laryngeal nerve; EBSLN: external branch of the superior laryngeal nerve.

^a^Fisher's exact test.

**Table 4:** Characteristics of patients stratified by thyroidectomy side (only for the control group).

	Left lobectomy	Right lobectomy	*P*
Age (years)	30.12 ± 6.41	30.32 ± 6.72	0.84
Male (%)	5 (7.1%)	2 (2.0%)	0.25^a^
Female (%)	79 (92.9%)	99 (98.0%)	
Volume of treated thyroid (mL)	15.06 ± 8.57 (2.91–27.59)	14.15 ± 8.43 (3.02–28.06)	0.48
Dominant nodule diameter (cm)	1.91 ± 0.83 (0.4–3.5)	1.84 ± 0.86 (0.4–3.5)	0.53
Transient RLN injury	10	4	0.04
Normal vocal cords condition	74	97	

RLN: recurrent laryngeal nerve.

^a^Fisher's exact test.
